# Role of p38 MAPK and STAT3 in lipopolysaccharide-stimulated mouse alveolar macrophages

**DOI:** 10.3892/etm.2014.2023

**Published:** 2014-10-15

**Authors:** AIHONG MENG, XIAOPENG ZHANG, YUNA SHI

**Affiliations:** 1 Respiratory Division, The Second Hospital of Hebei Medical University, Shijiazhuang, Hebei 050000, P.R. China; 2 Graduate School, Hebei Medical University, Shijiazhuang, Hebei 050000, P.R. China; 3 Department of Thoracic Surgery, Hebei Province General Hospital, Shijiazhuang, Hebei 050051, P.R. China

**Keywords:** lipopolysaccharide, MH-S cell line, mitogen-activated protein kinase, signal transducer and activator of transcription, SB203580

## Abstract

Excessive production of inflammatory mediators is an important feature of inflammatory lung disease. In macrophages, mitogen-activated protein kinase (MAPK) and signal transducer and activator of transcription-3 (STAT3) are crucial mediators for the production of proinflammatory cytokines. In the present study, the role of MAPK and STAT3 on tumor necrosis factor (TNF)-α and interleukin (IL)-10 production was investigated in mouse alveolar macrophages. The levels of TNF-α and IL-10 in lipopolysaccharide (LPS; 100 ng/ml)-stimulated MH-S cell lines were measured by an enzyme-linked immunosorbent assay, with or without p38 inhibitor (SB203580; 5, 10 or 15 μM) intervention. Phosphorylated STAT3 (p-STAT3) expression was examined by western blot analysis and immunocytochemistry following LPS stimulation for 15 or 30 min. Antibodies against STAT3 were used to verify comparable sample loading. Cells stimulated with LPS showed significantly increased levels of p-STAT3 protein (P<0.05) when compared with the baseline levels. TNF-α and IL-10 protein levels also increased following LPS stimulation (P<0.05). By contrast, treatment with the p38 inhibitor, SB203580, decreased the levels of p-STAT3, TNF-α and IL-10 (P<0.05) following LPS stimulation. SB203580 was shown to inhibit LPS-stimulated TNF-α expression (P<0.05) in a concentration-dependent manner, reaching significance at a concentration of 10 μM. However, the inhibition of IL-10 expression was not concentration-dependent. Therefore, LPS-stimulated overproduction of TNF-α and IL-10 is mediated at least partially by the MAPK pathway. Inhibition of p38 prevented LPS-induced STAT3 phosphorylation, indicating an interaction between the STAT3 and MAPK signaling pathways.

## Introduction

There are numerous inflammatory lung diseases (ILDs) that lack effective treatments aimed at the underlying causes, including adult respiratory distress syndrome, chronic obstructive pulmonary disease, idiopathic pulmonary fibrosis and asthma. ILDs are characterized by excessive inflammatory cell infiltration and the overproduction of inflammatory mediators, including tumor necrosis factor (TNF)-α, interleukin (IL)-1β and IL-6 (1–3).

ILD can be triggered by lipopolysaccharide (LPS), a cell wall component unique to gram-negative bacteria. A number of studies have shown that LPS can promote the development of ILD by stimulating immunocyte infiltration and inducing the production of inflammatory mediators (4–6).

Regulation of cytokine production in alveolar macrophages is mediated by the mitogen-activated protein kinase (MAPK) signaling pathway. This mechanism is crucial for the defense against bacterial infection; however, excessive production of these cytokines can cause lung injury. Therefore, reducing the release of inflammatory cytokines by inhibiting the MAPK pathway may alleviate lung injury (7).

In addition to the MAPK pathway, signal transducer and activator of transcription-3 (STAT3), a member of the cytoplasmic family of STATs, is also key to the development of ILDs, such as acute lung injury (ALI) (8). In previous studies, LPS was demonstrated to induce TNF-α expression in rat lungs, the spleen and alveolar macrophages from bronchoalveolar lavage fluid, and in these processes p38 MAPK and STAT3 signaling pathways were found to be involved (9–11).

In the present study, the interactions between the MAPK and STAT3 pathways and their roles in LPS-induced TNF-α and IL-10 production were investigated in cultured mouse alveolar macrophages (MH-S cells).

## Materials and methods

### Cells

MH-S cell lines purchased from the American Type Culture Collection (Manassas, VA, USA) were used for all the experiments. RPMI-1640 medium was purchased from Gibco Life Technologies (Grand Island, NY, USA). Fetal bovine serum (FBS), heat-inactivated and determined to be LPS-free, was purchased from Atlanta Biologicals, Inc. (Atlanta, GA, USA). LPS was purchased from Sigma-Aldrich (St Louis, MO, USA) and SB203580 (p38 MAPK inhibitor) was purchased from Promega Corporation (Madison, WI, USA). A rabbit polyclonal anti-tyrosine (Tyr 705) phosphorylated STAT3 (p-STAT3) antibody was used at a 1:500 dilution and was purchased from Santa Cruz Biotechnology, Inc. (sc-7993-R, Dallas, TX, USA).

### Culture and stimulation of MH-S cells

MH-S cells were grown in medium in 75-cm^2^ polystyrene tissue culture flasks. During log-phase growth, the cells were scraped off the flasks and the cell suspension was added to 24-well polystyrene tissue culture plates at 5×10^6^ cells/ml culture medium. The study included two experiments.

#### Experiment 1

After 24 h of growth at 37°C in 5% CO_2_, the cells were preincubated with SB203580 (5, 10 or 15 μM) for 20 min in 10% FBS-containing media, followed by the addition of LPS at a final concentration of 100 ng/ml. A blank control and LPS-stimulated control were included. Following stimulation for 1.5, 2, 4, 6 or 12 h, the supernatant was collected for subsequent TNF-α and IL-10 analyses.

#### Experiment 2

SB203580 (10 μM) was added to the cells 20 min prior to LPS (100 ng/ml) stimulation for 15 or 30 min. The cells were then collected and centrifuged (513 × g, 4°C, 10 min). The total protein extracts were stored at −80°C for examination of STAT3 phosphorylation by western blot analysis. Phosphorylation of STAT3 expression was also examined by immunocytochemistry.

### Examination of TNF-α, IL-10 and STAT3

Levels of TNF-α and IL-10 in LPS-stimulated MH-S cell lines were measured by enzyme-linked immunosorbent assays (RayBiotech,Inc.Norcross, GA, USA), with or without the p38 inhibitor, SB203580. Total STAT3 and p-STAT3 expression levels were examined by western blot analysis and immunocytochemistry. In brief, the procedure for the western blot analysis was the following: The cells were stimulated with LPS (100 ng/ml) for 15 or 30 min and the total cellular protein was extracted. To detect the phosphorylated STAT3, a rabbit polyclonal anti-tyrosine (Tyr 705) phosphorylated STAT3 antibody (Santa Cruz Biotechnology, Inc. sc-7993-R) was used as the primary antibody and anti-rabbit IgG secondary antibody (Beijing DingGuo biotechnology) was used. For the immunocytochemistry experiments the MH-S cells (5 × 10^5^/ml) were seeded on cover slips and cultured in a 6-well plate. The following day the cover slips were removed and fixed with 4 % paraformaldehyde solution at room temperature. In order to detect the p-STAT3 expression, the same antibodies described for the western blot analysis were used.

### Statistical analysis

Data are expressed as the mean ± standard deviation. The group differences were analyzed by one-way analysis of variance using SPSS 13.0 software (SPSS, Inc., Chicago, IL, USA). If significant, the data were further analyzed by the Student-Newman-Keuls test, where P<0.05 was considered to indicate a statistically significant difference.

## Results

### Changes in TNF-α and IL-10 levels in the MH-S cell supernatant

**Figure 1:**
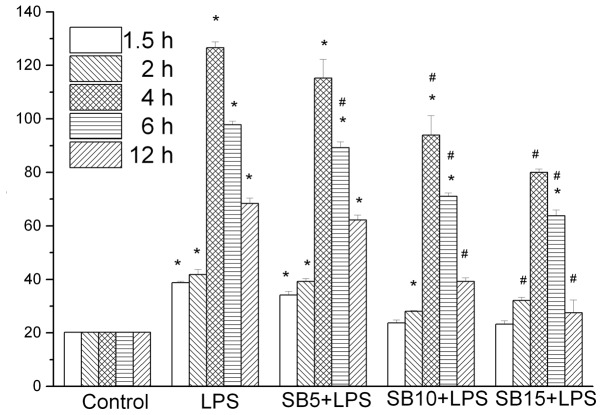
Effect of SB203580 (p38 mitogen-activated protein kinase inhibitor) on tumor necrosis factor-α (TNF-α) production in MH-S alveolar macrophages. The cells were pretreated with or without SB203580 (5, 10 or 15 μM) 20 min prior to lipopolysaccharide (LPS) stimulation. After incubation for 1.5, 2, 4, 6 or 12 h, the supernatants were obtained and the expression level of TNF-α was determined by an enzyme-linked immunosorbent assay. Results are expressed as the mean ± standard deviation for three independent experiments. ^*^P<0.05, vs. control group; ^#^P<0.05, vs. LPS group.

**Figure 2:**
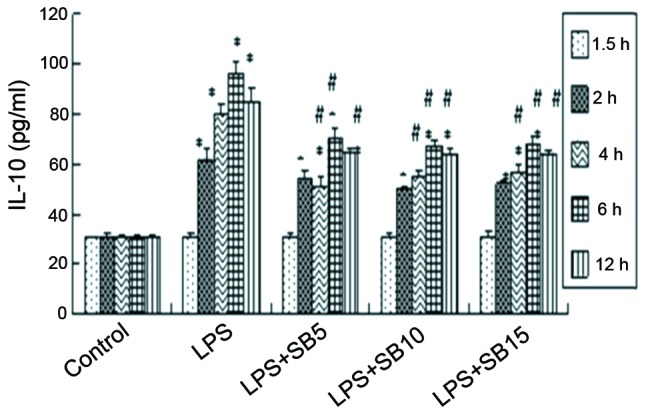
Effect of SB203580 (p38 mitogen-activated protein kinase inhibitor) on interleukin-10 (IL-10) production in MH-S cells. The cells were pretreated with or without SB203580 (5, 10 or 15 μM) 20 min prior to lipopolysaccharide (LPS) stimulation. Following incubation for 1.5, 2, 4, 6 or 12 h, the supernatants were obtained and IL-10 expression was determined by an enzyme-linked immunosorbent assay. Results are expressed as the mean ± standard error of the mean for three independent experiments. ^*^P<0.05, vs. control group; ^#^P<0.05, vs. LPS group.

TNF-α and IL-10 levels exhibited an increase at 90 min following LPS stimulation, reaching peak levels at 4 and 6 h, respectively. This trend was not observed in the control group (P<0.05). SB203580 treatment was shown to inhibit TNF-α in a dose-dependent manner (Fig. 1). Cells treated with 15 μM SB203580 produced significantly less TNF-α (P<0.05) compared with the SB203580 naive cells (Fig. 1). SB203580 treatment also inhibited IL-10; however, the dose-dependent inhibition was not significant. Furthermore, cells treated with 15 μM SB203580 produced significantly higher levels of TNF-α and IL-10 (Fig. 2) compared with the non-stimulated cells, indicating that pathways other than p38 may also mediate TNF-α production.

**Figure 3:**
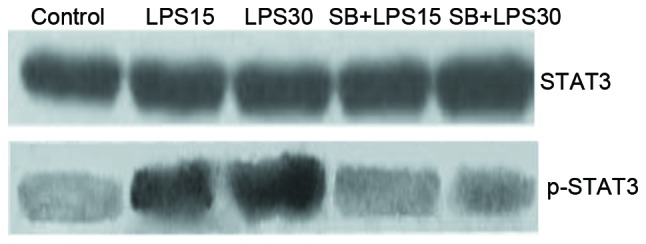
Effect of SB203580 (p38 mitogen-activated protein kinase inhibitor; 10 μM) on lipopolysaccharide (LPS)-induced phosphorylation of signal transducer and activator of transcription-3 (STAT3) activity in MH-S alveolar macrophages. The cells were stimulated with LPS (100 ng/ml) for 15 or 30 min. The cell lysates were collected and the phosphorylated-STAT3 (p-STAT3) activity was measured. Representative results of the two experiments are shown. The levels of p-STAT3 protein exhibited a marked increase 15 min after LPS induction (P<0.05) and peaked at 30 min after treatment. SB203580 treatment almost completely blocked STAT3 phosphorylation even at a concentration of 10 μM.

**Figure 4:**
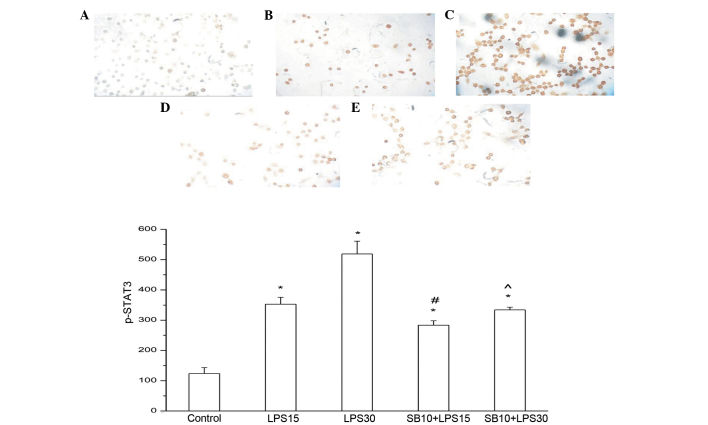
Immunocytochemical analysis of phosphorylated-signal transducer and activator of transcription-3 (p-STAT3) in the MH-S cells in the (A) control, (B) lipopolysaccharide (LPS) for 15 min, (C) LPS for 30 min (D) SB2035880 for 10 min + LPS for 15 min and (E) SB2035880 for 10 min + LPS for 30 min (diaminobenzidine stain; magnification, ×200). p-STAT3 expression increased significantly following LPS stimulation. Following the addition of SB203580, p-STAT3 expression decreased significantly. ^*^P<0.05, vs. control; ^#^P<0.05, vs. LPS at 15 min. ^P<0.05 vs. LPS30 min.

By contrast, the levels of p-STAT3 protein exhibited a significant increase 15 min after LPS induction (P<0.05), and peaked 30 min after treatment. SB203580 treatment almost completely blocked STAT3 phosphorylation, even at a concentration of 10 μM, indicating that p38 is a key regulator of STAT3 phosphorylation (Figs. 3 and 4).

## Discussion

The results of the present study demonstrated that in the MH-S cell supernatant, TNF-α levels increased following LPS stimulation and decreased following the addition of SB203580. The p-STAT3 expression levels increased in the LPS group, while SB203580 was found to inhibit the expression of p-STAT3, suggesting an interaction between p38 MAPK and STAT3.

ILDs are triggered by excessive inflammation, as exemplified by the overproduction of inflammatory mediators; however, the underlying mechanism is not fully understood. LPS-induced inflammation is an appropriate model since LPS principally acts on alveolar macrophages, causing a massive release of inflammatory cytokines. The cytokines and the cells that are involved in LPS-induced inflammation, have been basically defined; however, the mechanism by which these cytokines are expressed and regulated remains unclear (12,13). A previous study demonstrated that macrophage deficiency significantly attenuated TNF-α production in the lung tissues of mice (14).

The macrophage MAPK pathway is the key regulator of inflammation, regulating the gene expression of a variety of cytokines, and playing an important role in inflammation. Previous studies revealed that p38 MAPK was the basic signaling pathway in the regulation of LPS-induced TNF-α synthesis (9–11). LPS-induced TNF-α and IL-6 secretion, as well as neutrophil aggregation, protein leakage and bronchiostenosis, in the lungs of mice was found to be dependent on p38 MAPK signaling (15). Activation of extracellular signal-regulated kinase (ERK), p38, c-Jun N-terminal kinase (JNK) and nuclear factor-κB (NF-κB) was shown to be involved in LPS-induced TNF-α production in human monocytes. p38α is an important regulator of inflammatory responses, and p38α deficiency in macrophages has been shown to cause a significant inhibition in the production of LPS-induced TNF-α, IL-12 and IL-18 (16). However, a p38α deficiency was not shown to affect the LPS-induced activation of other major signaling pathways (NF-κB, JNK and ERK), nor the transcriptional activity of NF-κB (17). p38 MAPK modulates TNF-α transcription in LPS-activated primary human macrophages, which is mediated through p38 MAPK regulation of NF-κB. The regulation of NF-κB by p38 MAPK is cell-type dependent and this may have consequences for the anti-inflammatory efficacy of inhibitors of p38 MAPK (18). The present study clarified the role of p38 MAPK in LPS-induced inflammation and provided valuable data that may lead to effective treatment strategies for ILDs.

p38 MAPK, the primary intracellular signaling pathway that regulates LPS-induced TNF-α biosynthesis (19), can be blocked by a specific p38 MAPK inhibitor, SB203580. Under normal conditions, SB203580 is able to completely inhibit LPS-induced TNF-α expression by the RAW 264.7 mouse macrophage cell line; however, it lacks this inhibitory effect under hypoxic conditions (19).

Zhao *et al* (20) stimulated MH-S cells with LPS for different time periods and assessed the activation kinetics of ERK and p38 MAPK. In control alveolar macrophages, TNF-α levels increased while p38 activity was virtually undetectable. Following LPS stimulation, p38 was rapidly activated. Similarly, ERK was also potently activated in response to LPS stimulation, with kinetics similar to that of p38 activation. In the present study, MH-S cells were stimulated with LPS for 1.5, 2, 4, 6 and 12 h, and TNF-α production was shown to increase significantly, which is consistent with the results reported by Zhao *et al* (20). Although certain studies have demonstrated that p38 MAPK is a potential target in regulating TNF-α, the functions of p38 MAPK in different tissues should be further investigated (20–23). A previous study revealed that p38 MAPK and NF-κB were involved in LPS-induced TNF-α gene and protein expression in the rat spleen (11). The present study demonstrated that TNF-α expression in the supernatant was reduced by SB203580 in a dose-dependent manner, which indicated that in MH-S cells, SB203580 is able to decrease the secretion of TNF-α by inhibiting p38 MAPK, attenuating the LPS-induced inflammatory response.

The prognosis of ILD is hypothesized to depend on the balance between proinflammatory and anti-inflammatory cytokines. The STAT family is a key regulator of cytokine production, but there is limited knowledge of its role in mediating ILDs. STAT3 is an important member of the JAK-STAT pathway, and is widely expressed in a number of cell and tissue types. The activation of STAT3 has been implicated in the regulation of cell proliferation, differentiation, transformation, apoptosis and inflammation (24,25). STATs may be involved as a pathway in mediating ALI, regardless of the inciting factors (26). The STAT family has been reported to be expressed in ALI, while STAT3 has been shown to function as an anti-inflammatory protein with a protective role in ALI (27). STAT3 activation has been associated with suppressed inflammatory processes in experimental animals, murine myeloid cells and macrophage cell lines. Therefore, manipulation of STAT3 activation may facilitate the development of new pharmacological interventions in human inflammatory diseases. However, STAT3 activation is unable to directly regulate LPS signaling in human monocytes and may only represent part of the mechanism by which IL-10 suppresses TNF-α production in activated human monocytes (28). In a murine model of acute peritonitis, resident macrophages, but not other cell types, were shown to play a regulatory role in inflammation through a STAT3 signaling pathway (29). STAT3 appears to function as a repressor protein in this model of acute inflammation; however, STAT3 in other cell types may contribute to the production of TNF-α and macrophage inflammatory protein-2 (27). The present study demonstrated that p38 was a key regulator of STAT3 phosphorylation in mouse alveolar macrophages. STAT3 is activated by Tyr 705 and Ser 727 phosphorylation. Depletion of alveolar macrophages markedly reduced the extent of lung STAT3 activation and decreased the expression of a number of cytokines in the lungs, including IL-1β, IL-4, IL-6, IL-10 and TNF-α (30). Macrophages lacking STAT3 showed an abnormal activation phenotype, such as increased cytokine production as a result of endotoxin-induced inflammation.

One possible role of STATs in mediating lung injury is the upregulation of cytokine gene expression, with a previous study demonstrating that STAT3 is required for LPS-induced TNF-α expression in macrophages (31). The target genes of STATs may promote the pathological development of ALI, including cytokines, chemical factors, adhesion molecules and inflammatory regulators. STATs may increase LPS signaling molecules, including lipopolysaccharide binding protein and MD-2, indicating that STATs may expand the inflammatory response in the development of sepsis (32). Severgnini *et al* (26) reported that in LPS-stimulated mice, STAT3 was activated and TNF-α production increased. STAT3 signaling was reported to play a crucial role in the downregulation of TNF-α synthesis by human monocytes in the course of systemic inflammation *in vivo*, which suggests that STAT3 may be a potential molecular target for pharmacological intervention in clinical syndromes characterized by systemic inflammation (33). Blocking TNF-α signaling significantly attenuated LPS-mediated ALI, indicating that TNF-α was a plausible STAT target gene associated with ALI. LPS-induced MAPK activation, the production of endogenous IL-10, and STAT3 activation have been reported to play critical roles in the expression of suppressor of cytokine signaling 3 (SOCS3), which provides for feedback attenuation of cytokine-induced immune and inflammatory responses in macrophages (34). The results of the present study showed that TNF-α levels in the supernatant began to increase when the MH-S cells were stimulated for 90 min, while STAT3 phosphorylation increased with LPS stimulation for 15 min. These observations suggested that TNF-α may be regulated by STAT3, which is consistent with the findings of Severgnini *et al* (26).

Zhao *et al* (20) reported that the LPS-induced increase in TNF-α in MH-S cells occurred through the p38 MAPK pathway. The present study showed that the TNF-α levels increased following stimulation with LPS in MH-S cells, and decreased when SB203580 was added. However, further studies should investigate whether the TNF-α changes were dependent on STAT3, and whether STAT3 functions as a pro- or anti-inflammatory protein. Data on the activation and function of STAT3 and SOCS3 in the lungs during the acute inflammatory response are emerging, suggesting that these molecules may be potential targets for regulating pulmonary inflammatory responses (3).

Serine phosphorylation on Ser 727 by MAPK has been identified, and STAT may be the substrate of MAPK (35). In the present study, the rate of STAT3 Tyr 705 phosphorylation decreased when SB203580 was added to the MH-S cells, suggesting that STAT3 and p38 MAPK may be relevant to this process.

In a previous study (36), RAW 264.7 cells preincubated with the serum of burn rats treated with a topical p38 inhibitor showed a significantly lower TNF-α expression level following LPS stimulation when compared with the vehicle-treated group. Modulating p38 MAPK signaling in burn wounds was shown to reduce pulmonary microvascular injury and pulmonary edema (36). SB203580 can reduce the secretion of TNF-α through p38 MAPK; however, the compound is unable to inhibit the release of TNF-α in endotoxin shock mice and the subsequent mortality rate (37). Further study is required to clarify whether SB203580 can decrease the mortality rate of ALI.

In conclusion, the present study indicated that understanding the STAT pathway was critical to reveal the mechanisms of ILDs, as this pathway significantly influences immune regulation and the production of several important cytokines, and closely interacts with other signaling pathways. Understanding the signaling pathways relevant to specific conditions, including ILDs, may provide useful information for the development of novel therapeutic approaches.
